# Co_3_O_4_ as p-Type Material for CO Sensing in Humid Air

**DOI:** 10.3390/s17102216

**Published:** 2017-09-27

**Authors:** Svetlana Vladimirova, Valeriy Krivetskiy, Marina Rumyantseva, Alexander Gaskov, Natalia Mordvinova, Oleg Lebedev, Mikhail Martyshov, Pavel Forsh

**Affiliations:** 1 Faculty of Chemistry, Moscow State University, Moscow 119991, Russia; vladimirova.lagytina@gmail.com (S.V.); vkrivetsky@gmail.com (V.K.); gaskov@inorg.chem.msu.ru (A.G.); natalia.mordvinova@ensicaen.fr (N.M.); 2 Laboratoire CRISMAT, ENSICAEN-CNRS UMR6508, Caen 14050, France, oleg.lebedev@ensicaen.fr; 3 Faculty of Physics, Moscow State University, Moscow 119991, Russia; mmartyshov@mail.ru (M.M.); phorsh@mail.ru (P.F.); 4 National Research Center “Kurchatov Institute”, Moscow 123182, Russia

**Keywords:** cobalt oxide, p-type oxide semiconductor, gas sensor, CO sensor, humidity

## Abstract

Nanocrystalline cobalt oxide Co_3_O_4_ has been prepared by precipitation and subsequent thermal decomposition of a carbonate precursor, and has been characterized in detail using XRD, transmission electron microscopy, and FTIR spectroscopy. The sensory characteristics of the material towards carbon monoxide in the concentration range 6.7–20 ppm have been examined in both dry and humid air. A sensor signal is achieved in dry air at sufficiently low temperatures *T* = 80–120 °C, but the increase in relative humidity results in the disappearance of sensor signal in this temperature range. At temperatures above 200 °C the inversion of the sensor signal in dry air was observed. In the temperature interval 180–200 °C the sensor signal toward CO is nearly the same at 0, 20 and 60% r.h. The obtained results are discussed in relation with the specific features of the adsorption of CO, oxygen, and water molecules on the surface of Co_3_O_4_. The independence of the sensor signal from the air humidity combined with a sufficiently short response time at a moderate operating temperature makes Co_3_O_4_ a very promising material for CO detection in conditions of variable humidity.

## 1. Introduction

Resistive semiconductor gas sensors are widely used to control the quality of atmospheric air in industrial plants and in residential areas. They have many advantages: high sensitivity, small size, fast response, and low cost in mass production. n-Type metal oxides are widely used as sensitive materials [1], for example, SnO_2_ [2,3], ZnO [4,5,6], In_2_O_3_ [7,8], WO_3_ [9], TiO_2_ [10] and so on. One of the main disadvantages of these materials is their high sensitivity to humidity, which makes it difficult to use them in real outdoor conditions. The limitation of application of p-type semiconductor oxides CuO, NiO, Co_3_O_4_ etc. is mainly due to their high electrical resistance and lower sensitivity to gases in dry air in comparison with n-type oxides [11]. However, they are characterized by a higher concentration of chemisorbed oxygen [12], and exhibit high catalytic activity in oxidation reactions [13], which makes them promising materials for detecting gases in humid air.

Cobalt (II,III) oxide Co_3_O_4_ is a typical p-type semiconductor with a spinel structure. The Co^2+^ ions occupy one-eighth of the tetrahedral sites and Co^3+^ ions occupy a half of the octahedral sites [14]. The value of the band gap for cobalt oxide is 1.6–2.2 eV [11]. Among semiconductor oxides cobalt (II,III) oxide is one of the most active catalysts for the oxidation of carbon monoxide [15]. Its activity in CO oxidation is very close to that of noble metals [13]. The mechanism of interaction of carbon monoxide is considered in detail by the authors [13,14,16]. Briefly, the coexistence of Co^2+^/Co^3+^ ion pairs in the same material seems to be essential for this catalytic activity. In a simplified form, this was explained by the existence of the specific sites for O adsorption (Co^2+^-O-Co^3+^) and for CO adsorption (Co^2+^-CO), which requires a good balance between Co^2+^ and Co^3+^ species for an optimum activity in oxidation process [13].

There are a number of works devoted to the study of the sensor properties of Co_3_O_4_ to various gases in dry air—volatile organic compounds (VOCs): xylene [17], ethanol [18,19,20], methanol [20], acetone [17,21], as well as NH_3_ [22], NO_x_ [23], H_2_S [24], CO [16], etc. At the same time, only few articles have been devoted to the study of the sensor properties to CO of pure cobalt oxide in humid air [14,25].

In this article, the sensor properties of nanocrystalline cobalt oxide in CO detection have been investigated in dry (0% r.h.) and humid (20 and 60% r.h.) air. The temperature dependences of the sensor signal, calibration curves, and the values of response and recovery time have been obtained.

## 2. Materials and Methods

### 2.1. Preparation of Co_3_O_4_ Powder

To prepare the Co_3_O_4_, NH_4_HCO_3_ (3.95 g) was dissolved in deionized water (30 mL) and then slowly added to 1 M cobalt (II) nitrate solution (25 mL). The resulting precipitate was washed several times with deionized water and dried at 100 °C for 3 h. Finally, the powder was heated in air up to 500 °C (heating rate 5 °C/min) and annealed at this temperature for 24 h.

### 2.2. Characterization

The calcination temperature of precipitate was determined by thermogravimetric analysis–mass spectrometry (TG-MS) with heating rate 10 °C/min in an air atmosphere using a STA 409 PC Luxx thermal analyzer (Netzsch-Gerätebau GmbH, Selb, Germany) equipped with a QMS 403 C Aëolos quadrupole mass spectrometer (Netzsch-Gerätebau GmbH, Selb, Germany). The obtained cobalt oxide was characterized by X-ray diffraction (XRD) analysis on a DRON-4-07 diffractometer (Burevestnik, Moscow, Russia, CuK_α_ radiation, λ = 1.5406 Å), the crystallite size was calculated using the Scherrer formula. Measurement of the specific surface area and pore size analysis were carried out by the method of low-temperature nitrogen adsorption on the ASAP 2010 (Micromeritics, Norcross, GA, USA). Previously, the sample was evacuated at 300 °C to 0.4 Pa for 3 h. Based on the nitrogen adsorption isotherms obtained, the specific surface area, volume and average pore size were calculated by the Brunauer, Emmett and Teller (BET) and Barret, Johner and Halenda (BJH) methods.

The morphology, structure and chemical composition of the obtained cobalt oxide were studied at local scale by means of transmission electron microscopy (TEM). TEM and electron diffraction (ED) studies were carried out using a FEI Tecnai G2 30 UT microscope (FEI, Hillsboro, OR, USA) operated at 300 kV and having 0.17 nm point resolution and equipped with an EDAX EDX detector (EDAX, Mahwah, NJ, USA). High angle annular dark field scanning TEM (HAADF-STEM) images and EDX mapping were carried out using a JEM ARM 200F cold FEG double aberration corrected microscope (JEOL USA, Peabody, MA, USA) equipped with a CENTURIO EDX detector (JEOL USA, Peabody, MA, USA) and Quantum GIF (Gatan, Pleasanton, CA, USA).

The surface composition was investigated by Fourier transform infrared spectroscopy (FT-IR) in the wave number range of 4000–400 cm^−1^ region with a Frontier FT-IR spectrometer (PerkinElmer, Waltham, MA, USA) using the KBr pellet technique.

The type of conductivity of the cobalt oxide was determined using the Seebeck effect by applying a temperature gradient. The temperature gradient and the potential difference were measured by two thermocouples (copper/nickel-plated copper) and a digital voltmeter Expert 001 (Econics, Moscow, Russia) using the uniaxial 4-point geometry. The measurements were carried out on pressed Co_3_O_4_ pellets in the temperature range from room temperature to 80 °C in air.

To study the temperature dependence of the conductivity of cobalt oxide and for gas sensor measurements the sample was deposited as a paste onto a microelectronic chip consisting of Al_2_O_3_ insulator substrate with platinum heaters and contacts. The obtained thick films were dried in air at 50 °C for 48 h, heated to 200 °C (2 °C/min) and kept for 4 h to remove the organic binder. To investigate the CO gas sensing properties, the chip was placed in a test chamber with constant flux of 150 mL/min. The CO concentration (6.7, 10 and 20 ppm) in air was controlled by mass-flow controllers (Bronkhorst, Ruurlo, The Netherlands). The measurements were performed in the temperature range 60–300 °C. The required level of humidity (0, 20 and 60% r.h.) was provided by mixing two streams of dry air and humid air using a Cellkraft P-2 membrane humidifier (Cellkraft AB, Stockholm, Sweden). The background atmosphere was obtained from the pure air generation. The response S was calculated as $S=\frac{R_{CO}}{R_{air}}$, were $R_{CO}$ is the resistance of the film in the presence of CO, $R_{air}$ is the resistance of the film in pure air.

## 3. Results

**Figure 1:**
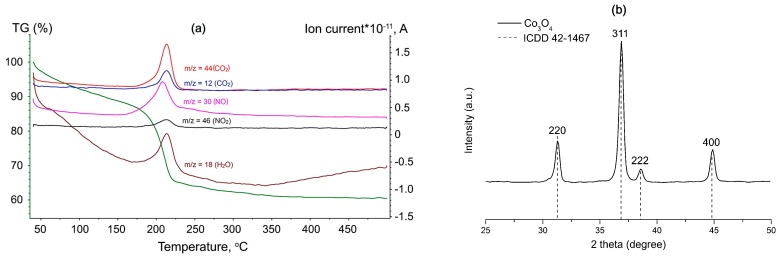
(**a**) TG-MS curves for of the dried precipitate; (**b**) X-ray diffraction patterns of Co_3_O_4_ nanoparticles.

The thermogravimetric analysis–mass spectrometry (TG-MS) was performed to determine the conditions for the precursor decomposition. Figure 1a shows the TG-MS curves of the carbonate precipitate dried at 100 °C. With the temperature increase the decomposition occurs in several steps. The first one is observed from room temperature to around 150 °C and brings a weight loss of 12%. It could be due to the dehydration process:

(1) $${\mathrm{CoCO}}_{3}\cdot xH_{2}O=\;{\mathrm{CoCO}}_{3}+xH_{2}O↑$$

Accordingly to the mass-spectral analysis of the gaseous products, the second step (25%, between 150 °C and 250 °C) is accompanied by the release of CO_2_ (*m*/*z* = 12, *m*/*z* = 44), NO (*m*/*z* = 30), NO_2_ (*m*/*z* = 46), and H_2_O (*m*/*z* = 18). So, this weight loss could be ascribed to the complex process including decomposition of un-reacted nitrate ions as well as reaction of the precipitate with oxygen:

(2) $$6{\mathrm{CoCO}}_{3}+{\;O}_{2}=\;2{\mathrm{Co}}_{3}O_{4}+6{\mathrm{CO}}_{2}↑$$

The third small weight loss (<3%) takes place at 350–500 °C temperature range and can be explained by the evaporation of residual water. According to the TG-MS data, heating to 500 °C and annealing at this temperature provides complete decomposition.

Figure 1b depicts the XRD pattern of obtained cobalt oxide. All the peaks correspond to the cubic Co_3_O_4_ phase with the spinel structure (ICDD 42-1467). The average crystallite size D_cr_ of the Co_3_O_4_ phase was estimated using the Scherrer formula as 26 ± 3 nm.

**Figure 2:**
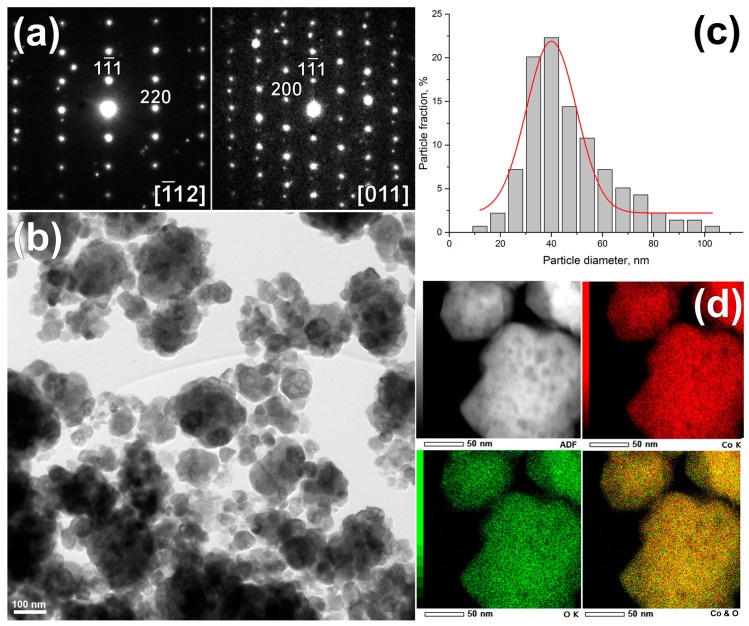
Characterization of Co_3_O_4_ nanoparticles by TEM: (**a**) typical ED patterns of the Co_3_O_4_ along main crystallographic zone axes $\bar{1}12$ and [011]; (**b**) bright field low magnification TEM image; (**c**) particle size distribution calculated from TEM images; (**d**) energy-dispersive X-ray spectroscopy (EDX) elemental maps of Co (red), O (green) and their mixture.

**Figure 3:**
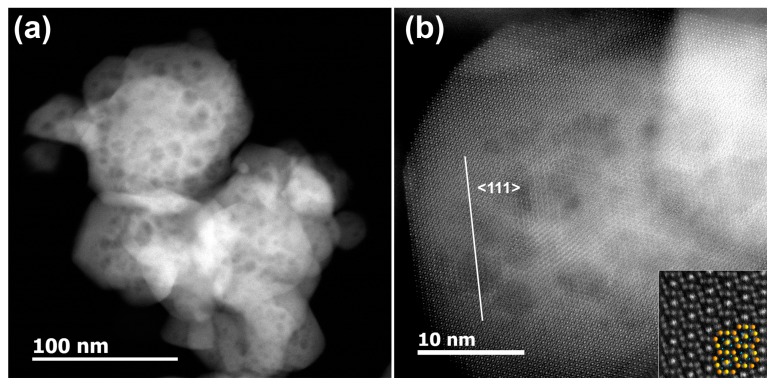
(**a**) Low-magnification and (**b**) high resolution HAADF-STEM images of Co_3_O_4_ nanoparticles viewing along the [011] zone axis. An enlarged image with overlapped structural model (Co—orange, O—blue) is given as inset.

The microstructure of cobalt oxide was investigated by TEM (Figure 2 and Figure 3). The selected area ED (SAED) patterns were taken from single nanoparticles along $\bar{1}12$ and [011] zone axis (Figure 2a) confirmed cubic Co_3_O_4_ spinel structure (Fd3m, a = 8.083 Å) determined by XRD and show perfectly crystallized structure. Since the size of selected aperture is slightly larger of single nanoparticle, other nanoparticles at the edge of the selected area aperture can contribute to SAED pattern resulting in appearance of extra spots. Figure 2b demonstrates that the sample consists of aggregated spherical particles with the broad size distribution from 15 to 100 nm. A size distribution was obtained from several TEM images from more than 150 particles manually and using the Image Pro Plus 6.0 software (Media Cybernetics Inc., Rockville, MD, USA). The mean particle diameter is 40 ± 7 nm (Figure 2c), which is in a good agreement with the average crystallite size D_cr_ calculated from XRD data. According to the EDX-STEM elemental maps shown in Figure 2d, oxygen and cobalt are distributed homogeneously within single particles.

**Figure 4:**
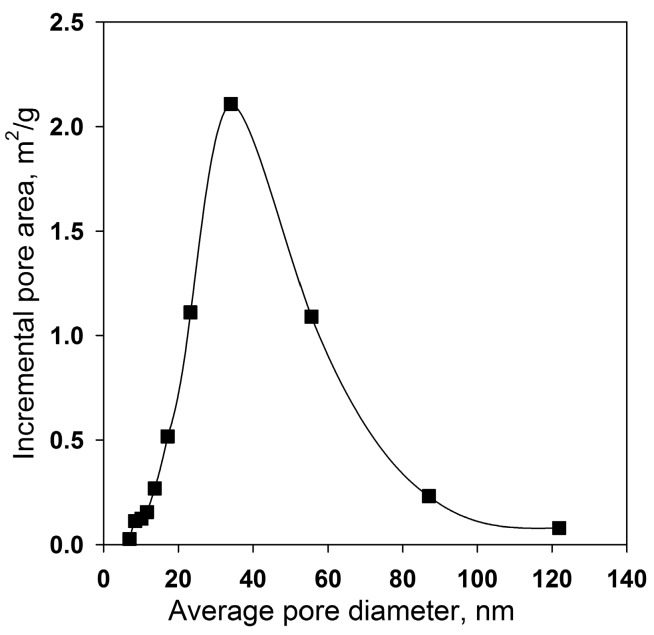
Pore size distribution calculated by BJH method.

From the HAADF-STEM image presented in Figure 3a one can clearly see that the prepared Co_3_O_4_ particles have a porous structure, with the mean pore diameter of around 3–7 nm due to the gases escaping during the decomposition process. However, it was determined by BET and BJH methods that the main contribution to the total surface area is made by pores with a diameter of 30–60 nm (Figure 4). This contradiction can be explained by the fact of agglomeration of the nanoparticles. In this case the pores with diameter of 30–60 nm are formed as a result of the contact between the particles in the agglomerates. The formation of agglomerates results in a low specific surface area of 8–13 m^2^/g. High resolution HAADF-STEM image of Co_3_O_4_ particle (Figure 3b) confirmed a good crystallinity of the material.

**Figure 5:**
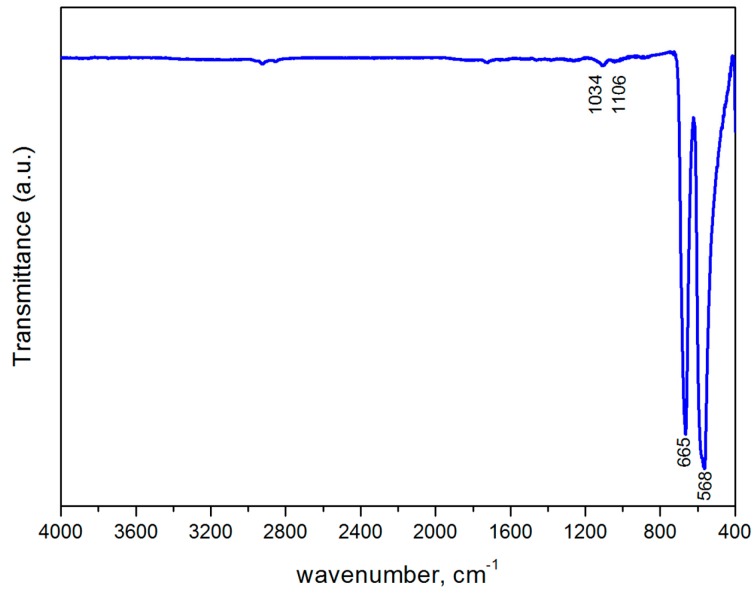
FT-IR spectra of Co_3_O_4_ nanoparticles.

The FT-IR spectra of nanocrystalline Co_3_O_4_ is shown in Figure 5. In the investigated region (4000–400 cm^−1^), the obtained spectra manifest the presence of two absorption bands at 568 and 665 cm^−1^ which originate from the stretching vibrations of the metal-oxygen bond (Co^3+^-O and Co^2+^-O) and confirm the formation of Co_3_O_4_ spinel oxide [24,26,27]. Peaks at 1034 and 1106 cm^−1^ are assigned to Co-O-H vibrations [28].

**Figure 6:**
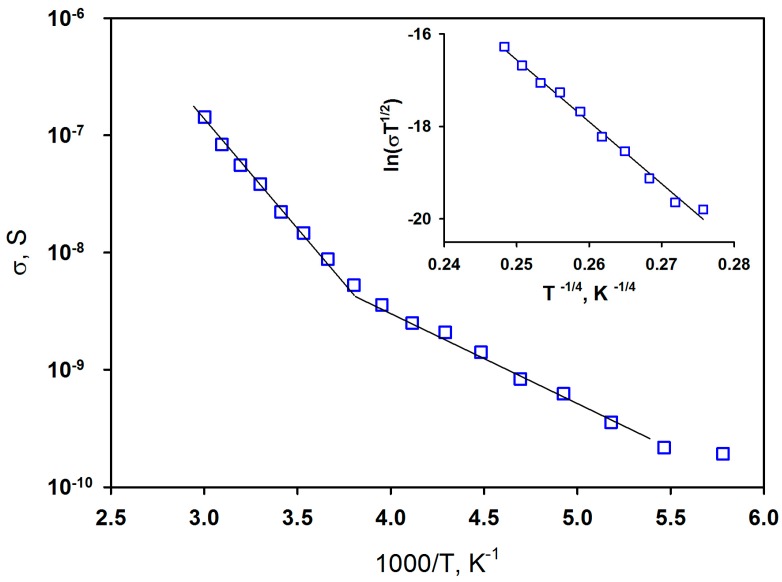
Temperature dependence of the conductivity of nanocrystalline Co_3_O_4_ thick film. Inset: temperature dependence of the conductivity in the Mott VRH model coordinates.

According to Seebeck effect measurements, the synthesized sample has a p-type conductivity. Figure 6 displays the variation of electrical conductivity of Co_3_O_4_ thick films (thickness 1.4 μm) with temperature, which demonstrates two different regions with activation energy of 0.35 eV (0–60 °C) and 0.15 eV (−100–0 °C). The energy 0.35 eV is consistent with the literature data obtained for pelleted powder samples [29,30] and can be attributed to the transition of an electron from the valence band to the level of the acceptor impurity. In the case of Co_3_O_4_ the presence of this level is caused by the cation vacancies, which are the main defects in the cobalt oxide structure at high oxygen pressure (in air) [31]:

(3) $$2O_{2}↔3V_{\mathrm{Co}}^{8/3^{\prime }}+\;8h^{\cdot }+4O_{O}^{X}.$$

This process is dominating in high temperature region. The temperature dependence of the conductivity in the interval *T* < 0 °C is discussed in [32] for Co_3_O_4_ films obtained by the CVD method. The authors assume that in this temperature range the conductivity mechanism has to be attributed to variable range hopping of holes (Mott VRH model [33]). Obviously, the data replotted in inset on Figure 6 as *σT*^1/2^ vs. *T*^−1/4^ demonstrate a linear relationship in the temperature range −100–0 °C that indicates the VRH carrier transport.

**Figure 7:**
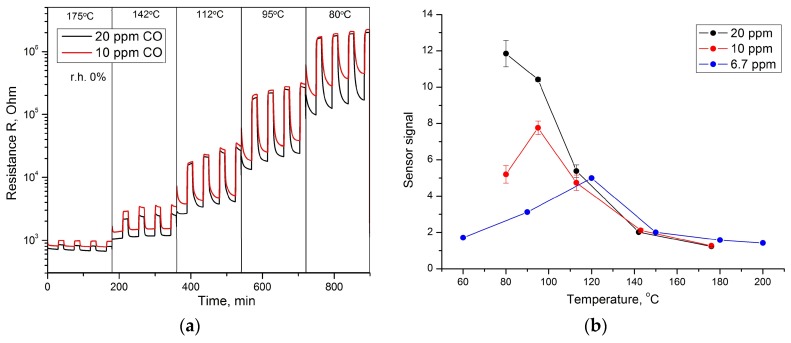
(**a**) Electrical response of Co_3_O_4_ to the periodic change of gas phase composition from dry air (30 min) to CO/air (15 min); (**b**) sensor response of the Co_3_O_4_ to CO (0% r.h.) as a function of temperature.

Figure 7a illustrates the electrical response of Co_3_O_4_ to the periodical change of gas phase composition from dry air to gas mixtures containing CO (10 or 20 ppm in dry air) in the temperature range of 80–175 °C. An increase in the electrical resistance in the presence of carbon monoxide is explained by the redox reaction:

(4) $$\beta \cdot {\mathrm{CO}}_{\left(\mathrm{gas}\right)}+O_{\beta \left(\mathrm{ads}\right)}^{-\alpha }↔\beta \cdot {\mathrm{CO}}_{2\left(\mathrm{gas}\right)}+\;\alpha \cdot e^{-}$$

where ${\mathrm{CO}}_{\left(\mathrm{gas}\right)}$ represent the reducing gas molecules in the gas phase, $O_{\beta \left(\mathrm{ads}\right)}^{-\alpha }$ is an atomic or molecular form of chemisorbed oxygen, ${\mathrm{CO}}_{2\left(\mathrm{gas}\right)}$ are the molecules of reaction products, e is an electron injected into the conduction band of the p-type semiconductor. With decreasing CO concentration, the temperature corresponding to the maximum sensor signal increases and is 80, 95, and 120 °C when detecting 20, 10, and 6.7 ppm CO, respectively (Figure 7b). Note, that at temperatures above 120 °C the sensor signal does not depend on the concentration of CO in dry air.

**Figure 8:**
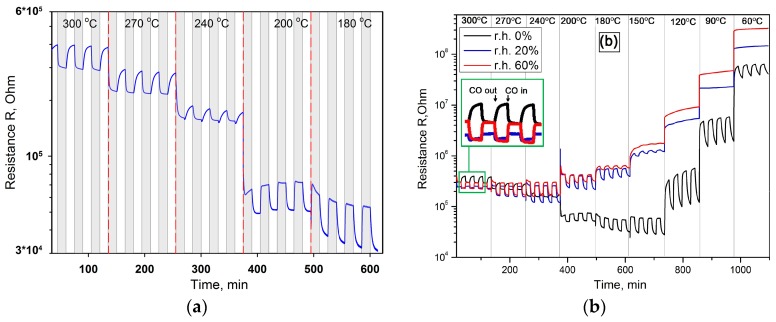
(**a**) Electrical response of Co_3_O_4_ to the periodic change of gas phase composition from 6.7 ppm CO/air (15 min) to pure air (15 min) (0% r.h.) in dry (**a**) and humid (**b**) conditions. On part (**a**) the time periods when CO is introduced into sensor chamber are marked by gray color.

**Figure 9:**
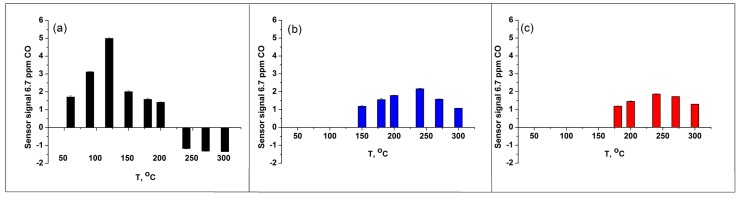
Bar chart showing the 6.7 ppm CO response of the Co_3_O_4_ sensor as a function of temperature at 0% r.h. (**a**), 20% r.h. (**b**), and 60% r.h. (**c**). The area of “negative” values in the temperature range 240–300 °C in dry air (part (**a**)) corresponds to n-type sensor behavior.

An exceptionally interesting fact is the appearance of inversion of the sensor signal during CO detection in dry air in the temperature range *T* = 240–300 °C: the sensor’s resistance decreases in the presence of reducing gas in reversible increases in pure air (Figure 8a). The increase in air humidity leads to changes in the interaction of cobalt oxide with CO (Figure 8b). The most significant difference is the complete disappearance of the sensory signal at low temperatures. Thus, at a humidity of 20% r.h., the change in the sensor resistance in the presence of CO can be registered at a temperature of 150 °C, an increase in the relative humidity of up to 60% r.h. leads to an increase in this temperature to 180 °C. Another crucial point is that there is no signal inversion in humid air (Figure 8b). The comparison of temperature dependencies of Co_3_O_4_ sensor signal to 6.7 ppm CO at different humidity levels is presented in Figure 9.

**Figure 10:**
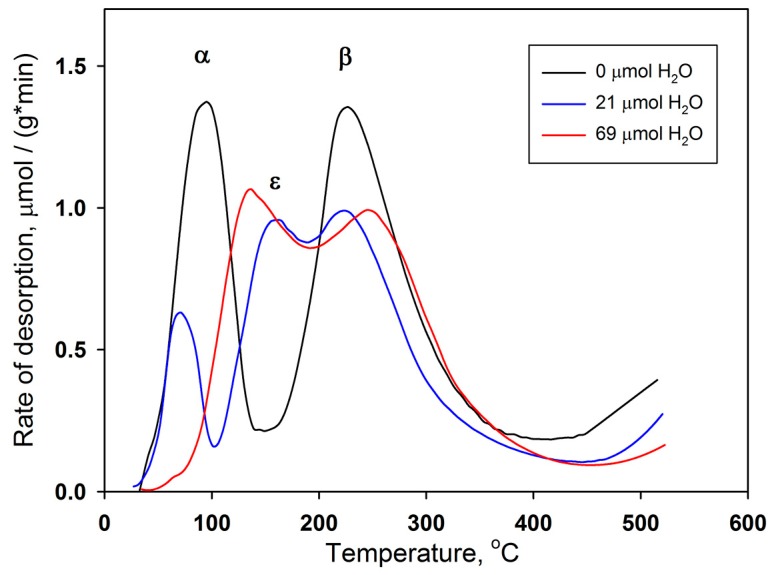
TPD curves of oxygen obtained after procedures consisting in oxygen adsorption after pre-adsorption of different quantity of water (0, 21 or 69 µmol/g Co_3_O_4_). Adapted from [34].

To discuss the results obtained, it is necessary to consider the specific features of the adsorption of CO, oxygen, and water molecules on the surface of Co_3_O_4_. Figure 10 presents the literature data of thermal programmed desorption (TPD) of oxygen from Co_3_O_4_ samples [34]. The TPD curves were obtained after procedures consisting in oxygen adsorption after pre-adsorbtion of different quantity of water (0, 21 or 69 µmol/g Co_3_O_4_). The TPD chromatogram obtained from the sample not treated by water vapor demonstrates two oxygen desorption peaks with the maxima at 90 and 227 °C and the ascent at *T* > 430 °C. Based on the spin unrestricted DFT+U calculations combined with the ab initio thermodynamic modeling and experimental data. Zasada et al. [35] revealed that in the oxygen pressure range of typical catalytic reactions, the most stable stoichiometric (100) surface accommodates the $\left[{\mathrm{Co}}^{O}-O_{2}-{\mathrm{Co}}^{O}\right]$ and $\left[{\mathrm{Co}}^{T}-O_{2}^{2-}-{\mathrm{Co}}^{O}\right]$ adducts at temperatures below 250–300 °C, where $\left[{\mathrm{Co}}^{T}\right]$ is cobalt cation in tetrahedral position, $\left[{\mathrm{Co}}^{O}\right]$ is cobalt cation in octahedral oxygen environment. So, the low temperature peak is attributed to the desorption of atomic-type oxygen species adsorbed on Co_3_O_4_ surface forming $\left[{\mathrm{Co}}^{O}-O_{2}-{\mathrm{Co}}^{O}\right]$ adduct (α oxygen). The pulse CO oxidation experiment effectuated in [35] demonstrated that low temperature CO oxidation occurs in the temperature range 40–180 °C with the maximum CO_2_ yield at 120 °C. Thus, it is α oxygen that is responsible for the formation of a sensory signal when detecting CO in dry air in low temperature interval 60–150 °C (Figure 9a).

A small amount of pre-adsorbed water (that corresponds to humid air atmosphere) brought about a drastic change in the TPD curves of oxygen (Figure 10) [34]. By increasing water pre-adsorption, the peak of α oxygen declined gradually and finally disappeared. At the same time, a new peak (ε oxygen) appeared and grew in size to almost that of the original α oxygen. The authors [34] assume that the partially negatively charged atomic oxygen species interacted with surface hydroxyl groups on a cobalt ion located at nearest neighbor through a hydrogen bond. The FTIR investigations [36] also demonstrated that the presence of humidity decreases the conversion of CO to CO_2_ and facilitates the formation of carbonates, which do not desorb from Co_3_O_4_ surface even at high temperature (300 °C). One can suppose, that these processes (transformation of active α oxygen into inactive ε oxygen and formation of carbonates) lead to the disappearance of gas sensitivity of Co_3_O_4_ in CO detection in low temperature interval.

The second oxygen desorption peak (β oxygen) (Figure 10) can be associated with the decomposition of $\left[{\mathrm{Co}}^{T}-O_{2}^{2-}-{\mathrm{Co}}^{O}\right]$ suprafacial adducts [35]. These reactive oxygen species can be responsible for the Co_3_O_4_ sensitivity to CO in the temperature interval 150–300 °C. However, at the temperature *T* > 200 °C an additional mechanism can be involved in the CO oxidation process. As follows from the detailed review [13], most authors have proposed a Mars–van Krevelen mechanism for CO oxidation over cobalt oxides. This indicates the participation of lattice oxygen in the CO oxidation reaction. From in situ FTIR spectroscopy, Lin et al. [37] concluded that two kinds of oxygen species could be involved in CO oxidation; a binuclear species (most likely superoxide) adsorbed on CoO_x_ and O anions of the Co_3_O_4_ spinel. Participation of lattice oxygen in the CO oxidation can lead to the formation of oxygen vacancies that can explain the inversion of the sensor signal in the high temperatures interval (*T* = 240–300 °C) in dry air (Figure 9a).

Formation of oxygen vacancies can be described as:

(5) $$O_{O}^{X}\to \frac{1}{2}O_{2}+{\;V}_{O}^{\cdot \cdot }+2e\prime$$

If in the surface layer of Co_3_O_4_ the electron concentration [n] begins to exceed the hole concentration [p], then the electrons become the main charge carriers ([n] > [p]) and the type of conductivity changes from p-type to n-type resulting in the inversion of sensor signal.

From TPD curves of oxygen (Figure 10) [34] one can see that, excluding the sample not treated by water vapor, β oxygen seems to be unchanged depending on the quantity of pre-adsorbed water. This correlate very well with the fact that the sensor signal to CO at 180–300 °C is nearly the same at 20 and 60% r.h. (Figure 9b,c). However, the absence of signal inversion in the humid air indicates that Mars–van Krevelen mechanism of CO oxidation does not realize in these conditions. Similar results, namely gas sensor response independent of relative humidity levels has been previously observed in H_2_S detection by p-type CuO nanowire sensor operated at 325 °C [38]. For CuO-based devices the mechanism of water and oxygen co-adsorption has been proposed in [39]. Considering this mechanism, the reaction with water vapor leads to the formation of terminal hydroxyl groups, and to the decrease in the concentration of chemisorbed oxygen species. This results in the growth of sensor baseline resistance, which was also observed in our experiments (Figure 8b).

**Figure 11:**
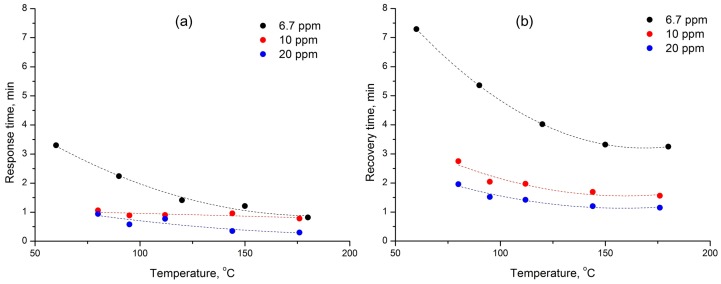
Values of response (**a**) and recovery (**b**) time as a function of temperature at 0% r.h.

**Figure 12:**
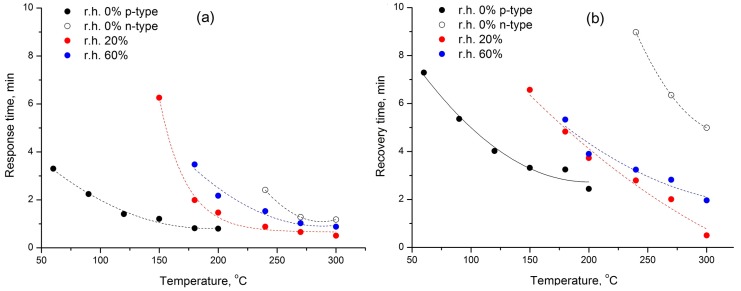
Values of response (**a**) and recovery (**b**) time as a function of temperature at 0, 20 and 60% r.h.

The values of response and recovery time obtained in dry air as a function of temperature are shown in Figure 11. The response time was defined as the time it takes for 75% of the sensor response change after CO introducing into the gas phase and the recovery time was the time needed for 75% of the sensor response change after CO removal from the gas phase. The values of the response and recovery times in dry air (0% r.h.) decrease as the CO concentration and the measurement temperature increase. Comparison of the data obtained at different humidity (Figure 12) reveals an agreement with the mechanism of CO oxidation. Thus, the values of response and recovery time for the processes, in which the formation of the sensor signal is due to the CO interaction with adsorbed oxygen (α oxygen in dry air or β oxygen in humid conditions) are close to each other and only shifted on a temperature scale. A significant increase in the recovery time in the case of inversion of the sensory signal, observed at a high measurement temperature in dry air (0% r.h., n-type response), is certainly due to the participation of the oxygen of the Co_3_O_4_ crystal lattice during the oxidation of CO by Mars–van Krevelen mechanism. One of the stages of this process is the filling of the oxygen vacancy by the oxygen atom, which is the limiting stage of the process as a whole.

## 4. Conclusions

Nanocrystalline p-type Co_3_O_4_ with a mean particle size of 30–40 nm and specific surface area of 8–13 m^2^/g has been prepared by the carbonate method and characterized in detail using XRD, ED, TEM and HAADF-STEM imaging and EDX analysis and FTIR spectroscopy.

The gas sensor properties of synthesized Co_3_O_4_ in CO detection were investigated in the temperature range 60–300 °C at different humidity levels 0, 20 and 60% r.h. In dry air the maximum sensor signal was observed in low temperature interval 80–120 °C. The increase in relative humidity results in the disappearance of sensor signal in this temperature range because of transformation of active oxygen species adsorbed on Co_3_O_4_ surface (α oxygen) into inactive ones (ε oxygen), and formation of thermally stable surface carbonates. The values of response and recovery time for the processes, in which the formation of the sensor signal is due to the CO interaction with adsorbed oxygen are close and do not depend on the humidity level.

In the temperature range *T* = 240–300 °C the inversion of the sensor signal was observed during CO detection in dry air. This fact is explained by the participation of the oxygen of the Co_3_O_4_ crystal lattice during the oxidation of CO by Mars–van Krevelen mechanism.

The sensor signal to CO at 180–200 °C is nearly the same at 0, 20 and 60% r.h. since CO molecules are oxidized by the reactive oxygen species (β oxygen) originated from $\left[{\mathrm{Co}}^{T}-O_{2}^{2-}-{\mathrm{Co}}^{O}\right]$ suprafacial adducts whose concentration is not greatly influenced by the quantity of pre-adsorbed water. This independence of the sensor signal from the air humidity combined with a sufficiently short response time at a moderate operating temperature makes Co_3_O_4_ a very promising material for outdoor and indoor CO detection.
